# Modeling Effects of L-Type Ca^2+^ Current and Na^+^-Ca^2+^ Exchanger on Ca^2+^ Trigger Flux in Rabbit Myocytes with Realistic T-Tubule Geometries

**DOI:** 10.3389/fphys.2012.00351

**Published:** 2012-09-10

**Authors:** Peter M. Kekenes-Huskey, Yuhui Cheng, Johan E. Hake, Frank B. Sachse, John H. Bridge, Michael J. Holst, J. Andrew McCammon, Andrew D. McCulloch, Anushka P. Michailova

**Affiliations:** ^1^Department of Pharmacology, University of California San DiegoLa Jolla, CA, USA; ^2^Florida State UniversityTallahassee, FL, USA; ^3^Department of Bioengineering, University of California San DiegoLa Jolla, CA, USA; ^4^Simula Research Laboratory, Center for Biomedical ComputingLysaker, Norway; ^5^Cardiovascular Research and Training Institute, University of UtahSalt Lake City, UT, USA; ^6^Department of Bioengineering, University of UtahSalt Lake City, UT, USA; ^7^Departments of Mathematics and Physics, University of California San DiegoLa Jolla, CA, USA

**Keywords:** Ca^2+^ signaling, L-type Ca^2+^ channel, Na^+^/Ca^2+^ exchanger, channel clustering, allosteric regulation, t-tubule, rabbit ventricular myocyte

## Abstract

The transverse tubular system of rabbit ventricular myocytes consists of cell membrane invaginations (t-tubules) that are essential for efficient cardiac excitation-contraction coupling. In this study, we investigate how t-tubule micro-anatomy, L-type Ca^2+^ channel (LCC) clustering, and allosteric activation of Na^+^/Ca^2+^ exchanger by L-type Ca^2+^ current affects intracellular Ca^2+^ dynamics. Our model includes a realistic 3D geometry of a single t-tubule and its surrounding half-sarcomeres for rabbit ventricular myocytes. The effects of spatially distributed membrane ion-transporters (LCC, Na^+^/Ca^2+^ exchanger, sarcolemmal Ca^2+^ pump, and sarcolemmal Ca^2+^ leak), and stationary and mobile Ca^2+^ buffers (troponin C, ATP, calmodulin, and Fluo-3) are also considered. We used a coupled reaction-diffusion system to describe the spatio-temporal concentration profiles of free and buffered intracellular Ca^2+^. We obtained parameters from voltage-clamp protocols of L-type Ca^2+^ current and line-scan recordings of Ca^2+^ concentration profiles in rabbit cells, in which the sarcoplasmic reticulum is disabled. Our model results agree with experimental measurements of global Ca^2+^ transient in myocytes loaded with 50 μM Fluo-3. We found that local Ca^2+^ concentrations within the cytosol and sub-sarcolemma, as well as the local trigger fluxes of Ca^2+^ crossing the cell membrane, are sensitive to details of t-tubule micro-structure and membrane Ca^2+^ flux distribution. The model additionally predicts that local Ca^2+^ trigger fluxes are at least threefold to eightfold higher than the whole-cell Ca^2+^ trigger flux. We found also that the activation of allosteric Ca^2+^-binding sites on the Na^+^/Ca^2+^ exchanger could provide a mechanism for regulating global and local Ca^2+^ trigger fluxes *in vivo*. Our studies indicate that improved structural and functional models could improve our understanding of the contributions of L-type and Na^+^/Ca^2+^ exchanger fluxes to intracellular Ca^2+^ dynamics.

## Introduction

In cardiac ventricular myocytes, invaginations of the cell membrane, known as t-tubules, promote rapid propagation of the action potential (AP) in the cell interior (Savio-Galimberti et al., 2008; Orchard et al., 2009; Smyrnias et al., 2010). The AP activates and modulates sarcolemmal Ca^2+^ fluxes, including fluxes through the L-type Ca^2+^ channels (LCC), Na^+^/Ca^2+^ exchangers (NCX), Ca^2+^ ATPase pumps, and background sarcolemmal Ca^2+^ leak (Bers, 2001). The entry of Ca^2+^ via LCC and NCX triggers the sarcoplasmic reticulum (SR) Ca^2+^ release via ryanodine receptors (RyRs). The SR provides Ca^2+^ for the troponin C (TnC) myofilament protein, thereby activating and regulating myocyte contraction (Bridge et al., 1990; Bers, 2001). Controversy, however, surrounds our understanding of whether the openings of LCCs can activate allosteric Ca^2+^-binding sites on NCX and how the clustering of LCCs, the allosteric catalysis of NCX, and cell surface shape modulate Ca^2+^ trigger flux controlling SR Ca^2+^ release (Sipido et al., 1997; Litwin et al., 1998; Egger and Niggli, 1999; Inoue and Bridge, 2003; Bers and Ginsburg, 2007; Cheng et al., 2010).

To investigate relationships between ion fluxes via LCCs and NCXs at voltages corresponding to the early AP plateau, Sobie et al. (2008) recently measured the time-dependent Ca^2+^ concentration profiles ([Ca^2+^]*_i_*) in isolated rabbit ventricular myocytes. Pharmaceutical disruption of SR activity enabled them to examine the contributions of LCC and NCX to Ca^2+^ trigger flux, which otherwise would have been masked by SR-bound fluxes from RyRs and the SR Ca^2+^ ATPase (SERCA). The study suggests that at positive voltages (*V*_m_ = +30 mV), the trigger flux of Ca^2+^ is greater than estimates from a simple summation of the fluxes through LCCs and Ca^2+^ entry via reverse NCX mode. The authors hypothesized that openings of LCCs increase local ([Ca^2+^]*_i_*) near the NCX protein complex, which activates Ca^2+^-binding sites on NCX and results in an increase of Ca^2+^ influx via the exchanger.

Mathematical modeling complements experimental studies by enabling the examination of Ca^2+^ signaling and excitation-contraction coupling (ECC) in cellular and sub-cellular environments. To this end, whole-cell models have suggested an intimate relationship between Ca^2+^, Na^+^, K^+^ ionic fluxes, and Ca^2+^ transient in rabbit ventricular myocytes (Shannon et al., 2004; Mahajan et al., 2008; Aslanidi et al., 2010). These models permitted examination of the contribution of various cellular components to the evolution of the Ca^2+^ transient under normal and certain pathological conditions. Furthermore, recent approaches have included approximate representations of the sub-cellular geometry to introduce spatial control of the predicted local and global Ca^2+^ transients (Langer and Peskoff, 1996; Michailova et al., 2002; Izu et al., 2006; Koh et al., 2006; Lines et al., 2006; Means et al., 2006; Lu et al., 2009; Soeller et al., 2009; Cheng et al., 2010, 2011, 2012a,b; Louch et al., 2010; Hatano et al., 2011, 2012; Sato and Bers, 2011; Yu et al., 2011; Hake et al., 2012). Lu et al. (2009) introduced a cylindrical representation of a t-tubule of rat ventricular myocytes. Cheng et al. (2010) extended this approach to use recently published t-tubule structures imaged in mice; these t-tubules displayed a large degree of branching unlike the simple cylindrical representation used by Lu et al.

In contrast to mice, the transverse tubular system (t-system) in rabbit ventricular myocytes exhibits a simple topology that more closely resembles the t-system of canine and human ventricular myocytes (Hayashi et al., 2009; Crossman et al., 2011; Sachse et al., 2012). Scanning confocal microscopy has yielded sub-micrometer resolution details of 3D structure of the rabbit t-system (Sachse et al., 2008, 2009) including wider t-tubule cross-sections compared to rodents, and structural variations such as constrictions and flattening (Savio-Galimberti et al., 2008). These large structural differences of the t-system, as well as variations in the density and expression of proteins, suggest that the local and global Ca^2+^ dynamics during myocyte contraction and relaxation may be species-dependent (Bers, 2001).

In this study we extended the model of Cheng et al. (2010), originally developed for rodent myocytes to incorporate structural data and model parameters specific to rabbit ventricular myocytes. We examined local and global Ca^2+^ dynamics under the influence of two applied transmembrane voltages (0 and +50 mV). We included equations describing the voltage-dependent background Ca^2+^ leak and the sarcolemmal Ca^2+^ ATPase pump (Lu et al., 2009). Furthermore, we evaluated a model of NCX allostery proposed by Weber et al. (2001) against experimental data (Sobie et al., 2008) and tested the hypothesis that the allosteric activation of NCX augments the global trigger flux for SR Ca^2+^ release in rabbits. Preliminary results of this work have been presented to the Biophysical Society in abstract form (Kekenes-Huskey et al., 2011).

## Materials and Methods

A summary of important model properties is presented here. Detailed methods, descriptions, definitions of variables and abbreviations, parameter values, and model equations are provided in the Supplementary Material. The model code is available to download from http://mccammon.ucsd.edu/smol and http://www.fetk.org, respectively.

### Geometric model

**Figure 1:**
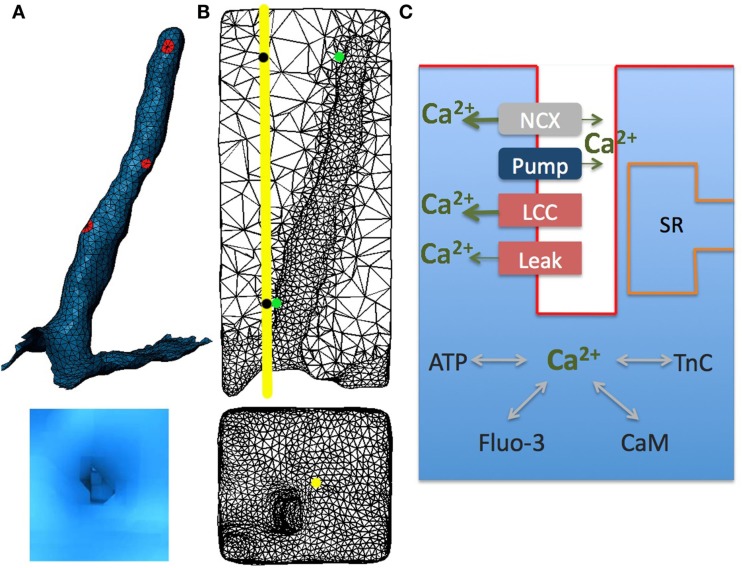
**Model geometry and diagram illustrating Ca^2+^ dynamics in rabbit ventricular myocytes with sarcoplasmic reticulum pharmacologically disabled**. **(A)** The cardiac sarcolemma, including external and t-tubule membranes were visualized using scanning confocal microscopy and labeled in Blender. Localized aggregates of L-type Ca^2+^ channels (red spots) were placed randomly within t-tubule membrane. **(B)** T-tubule mesh and its surrounding half-sarcomeres (upper panel); external membrane and t-tubule mouth (lower panel). **(C)** Schematic drawing of Ca^2+^ entrance and extrusion via the sarcolemma and Ca2^+^ buffering and diffusion inside the myocyte: LCC, L-type Ca2^+^ current; NCX, Na^+^/Ca^2+^ exchanger; Pump, membrane Ca2^+^ ATPase pump; Leak, background sarcolemmal Ca^2+^ leak; SR, sarcoplasmic reticulum; TnC, troponin C; CaM, calmodulin; ATP, adenosine triphosphate; Fluo-3, fluorescent dye. In all numerical experiments: LCC and NCX current densities were ninefold and threefold higher, respectively, in the t-tubule membrane; Ca^2+^ leak and pump were uniformly distributed along the sarcolemma; LCC clusters (diameter of (∼200 nm) had the same current density in the outer and t-tubular sarcolemma. The line-scan was positioned at 200 nm away from the t-tubule mouth [yellow line and yellow spot in **(B)**]. Local Ca^2+^ transients were extracted at two featured spots along the scanning line (black spots) and along the t-tubule membrane (green spots).

The model geometry was derived from the published structural data in rabbit ventricular myocytes (Sachse et al., 2008, 2009; Savio-Galimberti et al., 2008). The model contains one repeating unit inside the cell that includes: realistic surface sarcolemma; realistic t-tubule and its surrounding half-sarcomeres (Figures 1A,B). The surrounding half-sarcomeres were modeled as a rectangular-shaped box of 2.34 μm × 2.58 μm in the plane of external sarcolemma and 5.76 μm in depth.

The t-tubule diameter varied from between 0.39 and 0.62 μm and the length was ∼4.6 μm. The constrictions occurred every 1.87 ± 1.09 μm along the principal axis of the t-tubule and cross-sectional area of these constrictions was reduced to an average of 57.7 ± 27.5% (Savio-Galimberti et al., 2008). The volume of the model compartment was estimated to be 0.0282 pL. The compartment membrane area was 15.9 μm^2^ where the cell membrane within t-tubule was 7.8 μm^2^ and within the external membrane 8.1 μm^2^.

### Reaction-diffusion model

The effects of four exogenous and endogenous Ca^2+^ buffers (Fluo-3, ATP, calmodulin, and TnC) were considered (Figure 1C). The endogenous stationary buffer TnC was distributed uniformly throughout the cytosol, but not on the sarcolemma or the sub-sarcolemmal space (40–50 nm in depth; Shannon et al., 2004; Cheng et al., 2010). The free Ca^2+^ and mobile buffers (Fluo-3, ATP, and calmodulin) diffuse and react throughout the cytoplasm. The outer sarcolemma and sarcomere box faces were subject to reflective boundary conditions.

### Model currents

We examined the contributions of four ionic currents; *I*_LCC_, *I*_NCX_, *I*_Pump_, and *I*_Leak_. Formulation of *I*_NCX_ was presented as the product of an electrochemical (Δ*E*) and an allosteric factor (Allo; Weber et al., 2001). The maximum NCX rate value (*V*_max_NCX_ = 0.207 μM/ms) used here was from Shannon et al. (2004). The NCX allosteric constant (*K*_mCaAct_) was fitted to 0.29 vs. 0.256 μM in Shannon et al. (2004). The *K*_mCaAct_ value was adjusted to approximate the curvature of +50 mV whole-cell Ca^2+^ transient data from Sobie et al. (2008).

Immunohistochemical studies have demonstrated that most of the LCCs are concentrated in the t-tubules (from 3 to 9 times more than on the external sarcolemma; Scriven et al., 2000; Soeller et al., 2009). In this study, in agreement with the reported data, the LCC current density was assumed to be ninefold higher in the t-tubular membrane than in the outer cell surface. Measurements of Ca^2+^ sparks in rabbits and other species imply that a cluster of LCCs is likely involved in gating a cluster of RyRs (Lipp and Niggli, 1998; Bridge et al., 1999; Scriven et al., 2000, 2010; Takagishi et al., 2000; Inoue and Bridge, 2003; Altamirano and Bers, 2007; Dan et al., 2007; Poláková et al., 2008; Sobie and Ramay, 2009; Louch et al., 2010). Furthermore, 3D visualizations of fragments from rabbit ventricular myocytes have revealed that the majority of RyR clusters are adjacent to the t-system and that these clusters are irregularly distributed along t-tubules (Dan et al., 2007; Sachse et al., 2009). Thus here three patches (∼200 nm in diameter with the same LCC current density) were placed randomly along the t-tubule (Figure 1A). Data also suggest a smaller number of RyR clusters to co-localize with LCCs on the external membrane (Franzini-Armstrong et al., 1999; Chen-Izu et al., 2006; Dan et al., 2007; Baddeley et al., 2011; Sachse et al., 2012). Dan et al. (2007) measured ∼2 μm longitudinal periodicity of RyR clusters on the cell surface and Sachse et al. (2009, 2012) observed irregular cluster distributions in transverse sheets. As surface RyR cluster positions with respect to t-tubule mouth remain controversial, in our model we assumed that the LCC current density was continuous along the outer membrane. Because in adult rabbits a lesser degree of clustering for NCX has been demonstrated (Lin et al., 2009; Gershome et al., 2011), we also assumed a continuous NCX distribution with a threefold higher density along the t-tubule (Neco et al., 2010). Here Ca^2+^ pump and leak currents were uniformly distributed along the model surface (Shannon et al., 2004; Lu et al., 2009; Brini and Carafoli, 2011).

**Figure A1:**
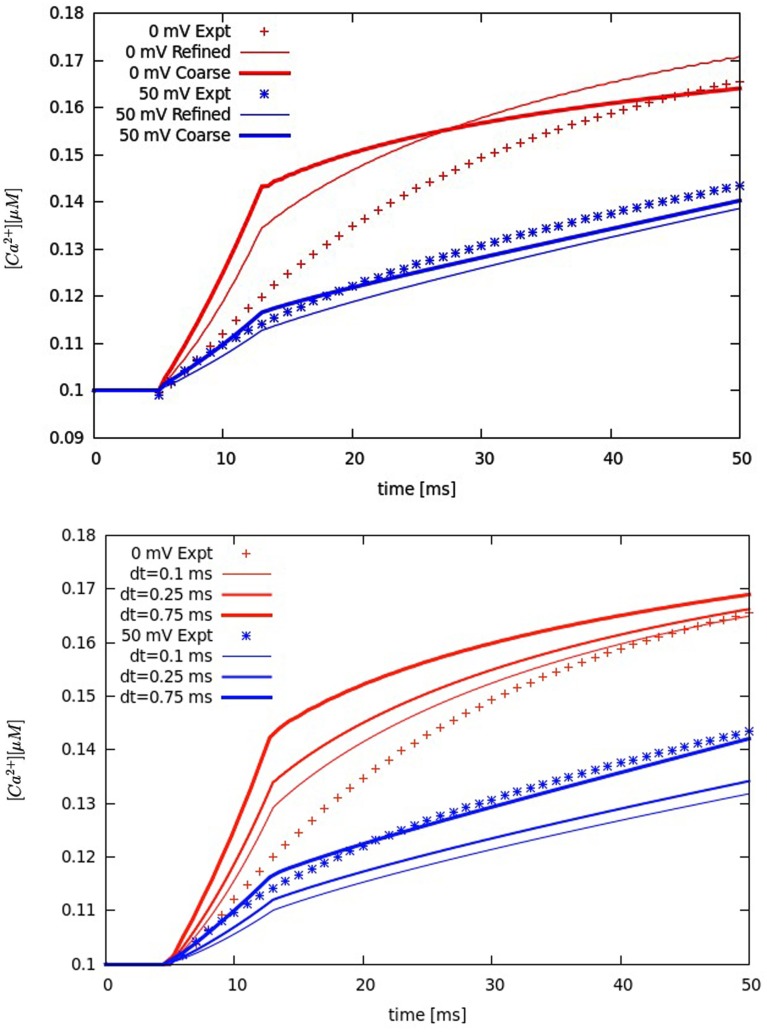
**Solution convergence analysis**. Upper panel: refining the original mesh. Lower panel: changing the original time step of 0.5 ms. Global Ca^2+^ transients experimentally measured (*symbols*). Membrane voltage at 0 mV (*red lines* and *symbols*) and at +50 mV (*blue lines* and *symbols*). [Na^+^]*_i_* 10 mM.

In this study, each simulation started with basal cytosolic ([Ca^2+^]*_i_*) of 0.1 μM, and buffers in equilibrium. The extracellular Ca^2+^ and Na^+^ concentrations were 2 and 140 mM, respectively, and remained constant. The voltage-clamp protocols (holding potential −50 mV, voltage step to 0 or +50 mV for 200 ms) were derived from data in rabbits (Sobie et al., 2008). Each current density (*I*_LCC_, *I*_NCX_, *I*_Pump_, and *I*_Leak_) was converted to Ca^2+^ flux (see Eq. A7, Supplementary Material) by using the experimentally suggested surface to volume ratio (*C*_m_/*V*_cell_ ∼ 4.54 pF/pL) in adult rabbit ventricular myocytes (Bers, 2001). To ensure the total Ca^2+^ flux through L-type channels to be the same as measured at given voltage, the model protocols for whole-cell LCC current were fitted vs. data reported in rabbits (Sobie et al., 2008). To justify the model predictions a solution convergence analysis has been performed. Multiple tests, including refining the original mesh or changing the original time-step size for integration (0.5 ms), demonstrated that the used mesh and time step were correctly chosen (see Figure A1 in Appendix).

## Results

### Global Ca^2+^ signals

**Figure 2:**
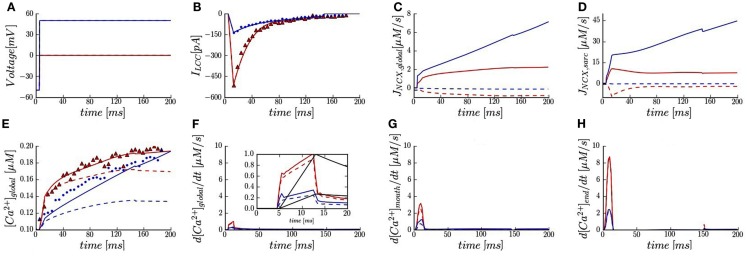
**Membrane currents, calcium signals, and trigger fluxes in the presence of 50 μM Fluo-3**. **(A)** Voltage-clamp protocols. **(B)** Whole-cell LCC current fitted and plotted (*solid lines*) vs. data reported in rabbits with SR blocked (*triangles* and *dots*). **(C,D)** Predicted global and sub-sarcolemmal Na^+^/Ca^2+^ fluxes. **(E)** Global Ca^2+^ transients (*solid* and *dash lines*) vs. experimentally measured (*triangles* and *dots*). **(F)** Predicted trigger fluxes from global Ca^2+^ transient. Inset shows normalized whole-cell LCC currents (*black lines*) and normalized trigger fluxes at 0 and +50 mV. **(G,H)** Local Ca^2+^ trigger fluxes at 1.5 and 5.3 μm (*green spots* in Figure 1B). Membrane voltage at 0 mV (*red lines* and *symbols*) and at +50 mV (*blue lines* and *symbols*). [Na^+^]*_i_* 10 mM (*solid lines*) and [Na^+^]*_i_* 0 mM (*dash lines*).

We validated the model against whole-cell measurements of the L-type Ca^2+^ current and Ca^2+^ transient at two voltages (0 and +50 mV) in the presence of 50 μM Fluo-3 and [Na^+^]*_i_* of 10 mM (Sobie et al., 2008). The voltage-clamp protocols and LCC currents are shown in Figures 2A,B. Global NCX time-courses are shown in Figure 2C. Calcium pump and leak fluxes are not shown here due to their small contribution. The model predicts at 0 mV a steep increase in global [Ca^2+^]*_i_* that tapers off at ∼40 ms and converges to 0.19 μM as shown experimentally (Figure 2E). At +50 mV, a more gradual accumulation of Ca^2+^ is predicted until converging to ∼0.2 vs. 0.19 μM experimentally. These results indicate that our model is a reliable representation of whole-cell Ca^2+^ dynamics as measured in rabbit ventricular myocytes (Sobie et al., 2008).

### Global and sub-sarcolemmal NCX fluxes

In agreement with experiment (Sobie et al., 2008), the model predicts that global NCX flux (*I*_NCXglobal_; computed by averaging local [Ca^2+^]*_i_* levels for the entire compartment) is in reverse mode at both applied voltages with 10 mM [Na^+^]*_i_* (Figure 2C). For [Na^+^]*_i_* of 0 mM, the NCX reverse mode is inactivated and an outward Ca^2+^ flux is predicted. The contribution to the global Ca^2+^ transient due to NCX was largest at +50 mV and [Ca^2+^]*_i_* monotonically increases with time.

Figure 2D shows NCX flux calculated by averaging local [Ca^2+^]*_i_* levels in the sub-sarcolemmal space (*I*_NCXsarc_). In the presence of 10 mM [Na^+^]*_i_*, our results demonstrate that: (1) the overall scale of *I*_NCXsarc_ was ∼5.6-fold greater than *I*_NCXglobal_; (2) the increase in *I*_NCXsarc_ during *I*_LCC_ upstroke relative to *I*_NCXglobal_ was much faster; (3) while *I*_NCXglobal_ monotonically increased over the entire simulation, local peaks in *I*_NCXsarc_ are predicted at 15 ms. Moreover, in absence of [Na^+^]*_i_*, the model predicts a sharp reversal in *I*_NCXsarc_ at 0 mV, whereas *I*_NCXsarc_ had a more gradual and monotonically decreasing flux.

### Global and local Ca^2+^ trigger fluxes

The whole-cell trigger flux reported by Sobie et al. (defined as *dF*/*dt*_max_) quantifies the maximum inward Ca^2+^ flux. By normalizing *dF*/*dt*_max_ and *I*_LCC_ to 1.0, it was suggested that the relative contribution of global NCX flux can be estimated (Sobie et al., 2008). Here we first calculated our global trigger flux (*d*[Ca^2+^]_global_/*dt*, Figure 2F) from the predicted global Ca^2+^ transient. We then normalized *I*_LCC_ and *d*[Ca^2+^]_global_/*dt* based on their maximum values, which both occur at 15 ms for 0 mV. In the presence of 10 mM [Na^+^]*_i_*, our results show that at +50 mV, *d*[Ca^2+^]_global_/*dt*_max_ is ∼30% of the value at 0 mV while experimental value is reported as 45%. Furthermore, *I*_LCC_ constitutes 70% of the trigger flux at +50 mV in comparison to 50% measured (Figure 2F Inset vs. Figure 2 in Sobie et al.). For zero [Na^+^]*_i_*, in agreement with experiment (Figure 2F Inset vs. Figure 3 in Sobie et al.), there is negligible impact on *d*[Ca^2+^]_global_/*dt*_max_ at 0 mV (∼10% reduction) and at +50 mV, a ∼30% reduction in *d*[Ca^2+^]_global_/*dt*_max_ is predicted relative to a measured 50% decline.

The model also provides estimates of local Ca^2+^ trigger fluxes within the sub-sarcolemmal space. At 0 mV, the model predicts an approximately threefold increase in *d*[Ca^2+^]/*dt*_max_ near the t-tubule mouth relative to the global trigger flux (Figures 2F,G). Moreover, the local *d*[Ca^2+^]/*dt*_max_ at the cell exterior and distal end of t-tubule (adjacent to LCC cluster) were very different: 3 vs. 9 μM/s at 0 mV; 1.3 vs. 2.5 μM/s at +50 mV (Figures 2G,H). At +50 mV a roughly 30% drop in *d*[Ca^2+^]_mouth_/*dt*_max_ was predicted in the absence of [Na^+^]*_i_*.

### Local Ca^2+^ signals

**Figure 3:**
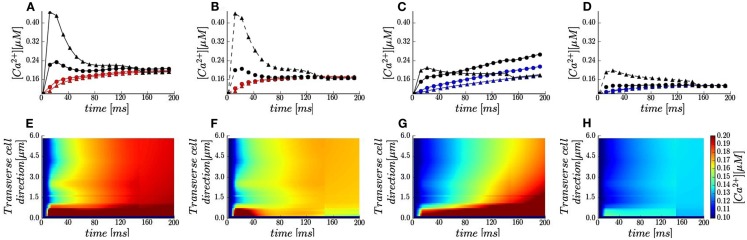
**Local calcium signals in the presence of 50 μM Fluo-3. (A–D)** Local Ca^2+^ transients taken at two featured spots along the scanning line (*red* and *blue lines* and *symbols*) and the t-tubule membrane (*black lines* and *symbols*): *circle* −1.5 μm; *triangles* −5.3 μm. Membrane voltage at 0 mV [*red* and *black lines* in **(A,B)**] and at +50 mV [*blue* and *black lines* in **(C,D)**]. [Na^+^]*_i_* 10 mM (*solid lines*) and [Na^+^]*_i_* 0 mM (*dash lines*). **(E–H)** Calcium concentrations visualized as line-scan images in transverse cell direction for the cases described in **(A–D)**.

In Sobie’s et al. (2008) experiment in rabbits the sarcolemma were not labeled and the fluorescence signal was recorded along the single scan lane at unknown orientations (Sobie personal communication). Due to these experimental limitations, we assumed the scanned line oriented in transverse cell direction (Figure 1 in Sobie et al., Figure (1B *yellow line*) to probe contributions of the realistic surface and t-tubule shape to local Ca^2+^ profiles. The calculated line-scan images and local Ca^2+^ time-courses are shown in Figures 3A–H. In agreement with experiment with 10 mM [Na^+^]*_i_* non-uniform Ca^2+^ distributions for both voltages are predicted (Figures 3E,G vs. Figure 1 in Sobie et al.). The contribution of a LCC cluster located ∼2 μm away from upper surface is evident as a spike in the line-scan images. At +50 mV a slight Ca^2+^ gradient is predicted in transverse cell direction (Figure 3G). In addition, results in Figures 3A,D show that local Ca^2+^ transients at both locations along the scanning line (1.5 μm *red* and *blue circles*; 5.3 μm *red* and *blue triangles*) follow the same trends as the global Ca^2+^ transients.

The model also predicts that local Ca^2+^ transients in the proximity of a t-tubule (Figure 1B *green spots*) differ considerably with respect to local cytosolic Ca^2+^ transients. Adjacent to the LCC cluster located at the t-tubule distal end (Figure 1A), the local Ca^2+^ transients closely resembles the *I*_LCC_ profiles with times to peak ∼15 ms followed by a decay to the cytosolic [Ca^2+^]*_i_* by 200 ms (Figures 3A–D *black triangles*). Toward the cell surface, the trends depend on the applied voltage (Figures 3A–D *black circles*). At 0 mV, the [Ca^2+^]*_i_* peak at the cell surface is nearly half of the transient at the LCC cluster and occurs ∼10 ms later. At +50 mV, the sub-membrane [Ca^2+^]*_i_* initially increases faster than the cytosolic analog (for *t* < 20 ms) and thereafter [Ca^2+^]*_i_* increases at the same rate as local cytosolic. We should mention here, that such local Ca^2+^ signals are difficult to resolve experimentally due to optical blurring and noise. Thus, our modeling study is one more example that computational models may serve as powerful tools for prediction and analysis on how local Ca^2+^ dynamics is regulated.

The most compelling evidence of the voltage and [Na^+^]*_i_*-influenced [Ca^2+^]*_i_* transient is shown in Movie S1 in Supplementary Material. Consistent in all movies is the predicted large and steep Ca^2+^ gradient in the narrow sub-sarcolemmal region. In these movies, the LCC clusters are clearly evident as localized regions of enhanced local [Ca^2+^]*_i_*. Comparison of the 0 vs. +50 mV cases (*top* and *bottom rows*) demonstrates that at both voltages [Ca^2+^]*_i_* increases heterogeneously in transverse cell direction as suggested by experiment (Sobie et al., 2008). A spontaneous increase in sub-membrane [Ca^2+^]*_i_* at +50 mV propagating within the cell late in the simulation is predicted also, while initiation and propagation of Ca^2+^ gradients at both voltages has not been observed during experiment. Additional interesting results in the presence 10 mM [Na^+^]*_i_* are that: (1) Ca^2+^ gradient traveling from the external membrane to the cell interior is predicted at both voltages when LCCs were continuously distributed along the t-tubule (Movie S2 in Supplementary Material, *right column*); and (2) lesser inwardly propagating Ca^2+^ gradient was observed at +50 mV when using the non-allosteric model of NCX (e.g., Allo = 1; Movie S3 in Supplementary Material, *lower left panel*).

The interaction between adjacent Ca^2+^ release units (CRU, local functional unit where LCC and RyR clusters reside) has been suggested to be critically dependent on the distance between one unit and its immediate neighbor (Franzini-Armstrong et al., 1999; Scriven et al., 2000; Izu et al., 2006; Dan et al., 2007; Hayashi et al., 2009). To test this, we held fixed the LCC cluster placed ∼2 μm away from upper surface while placing the other two clusters at various inter-cluster spacings. These spacings include ∼0.57, ∼0.8, 1.07, and ∼1.8 μm, whereas 0.78 ± 0.21 μm for the nearest end-to-end CRU distance was measured in rabbits (Dan et al., 2007; Savio-Galimberti et al., 2008). We found that predicted spatial [Ca^2+^]*_i_* distributions are highly sensitive to the spacing between LCC clusters (Movie S4 in Supplementary Material).

Finally, to gain further insights on the role of NCX flux in regulating local Ca^2+^ dynamics, we tested how the changes in NCX allosteric constant (*K*_mCaAct_) and maximum exchanger rate (*V*_max_NCX_) affect the results. Our data indicate that local Ca^2+^ profiles are highly sensitive to *K*_mCaAct_ and *V*_max_NCX_ alterations (Movie S5 in Supplementary Material vs. Movie S1 in Supplementary Material *left lower panel*).

## Discussion

### Model geometry and L-type Ca^2+^ channels clustering

Numerical studies of Ca^2+^ signaling in rodent cardiomyocytes that consider non-trivial geometries have shown that the spatial Ca^2+^ dynamics depends on myocyte micro-structure, including the branching of t-tubules (Lu et al., 2009; Soeller et al., 2009; Cheng et al., 2010; Hake et al., 2012; Hatano et al., 2012). Therefore, we hypothesized that details specific to rabbits, e.g., the linear t-tubule structure with varying diameter and eccentricity, provide unique control of the Ca^2+^ transient (Sachse et al., 2008; Savio-Galimberti et al., 2008). Our studies indicate that these factors may contribute to a spatially non-uniform [Ca^2+^]*_i_* distribution (Sobie et al., 2008; Bridge and Sachse, personal communication) while in rats, the measured [Ca^2+^]*_i_* profiles were more evenly distributed with SR activity disabled (Cheng et al., 1994).

In this study, we also assumed a clustered distribution for LCCs along the t-tubule based on a random distribution. The model predicts that clustering of LCCs resulted in more uniform [Ca^2+^]*_i_* profiles along the transverse cell direction relative to a continuous LCCs distribution. This greatly reduced the amplitude of the outer sarcolemmal compared to the continuous LCCs distribution (Movie S2 in Supplementary Material). New findings are also that local Ca^2+^ levels are highly sensitive to LCC cluster positions along the t-tubule (Movie S4 in Supplementary Material). In the model LCCs were also uniformly distributed on the cell surface, yet RyR distribution data suggest a small number of LCC clusters may also co-localize on the external membrane (Chen-Izu et al., 2006; Dan et al., 2007; Sachse et al., 2009, 2012). Thus, we placed two LCC clusters within the surface membrane near the t-tubule mouth but found that the LCC cluster placement intensified the under-membrane [Ca^2+^]*_i_* non-uniformity (*data not shown*). We speculate here that the localization of two LCC clusters along the cell surface within the half-sarcomere micro-domain probably overestimates the outer sarcolemma contribution (two outer LCC clusters vs. three t-tubule LCC clusters). In addition, our assumption of identical current profiles for each LCC cluster may be inappropriate, since the cluster shape and size, and the number of L-type channels involved in single spark triggering will certainly modulate the local current and local [Ca^2+^]*_i_* profiles (Franzini-Armstrong et al., 1999; Chen-Izu et al., 2006; Hayashi et al., 2009; Louch et al., 2010; Scriven et al., 2010). Although our concept of LCC clustering is quite rudimentary, this is a first attempt to examine the effects of LCC clusters, incorporated in more realistic membrane shapes, on local Ca^2+^ dynamics in light of evidence that LCC clusters exist in junctional clefts and form part of the couplon. A more appropriate description may require modeling LCC patches comprised of LCC “sub-clusters,” given their suggested involvement in the triggering of RyRs in the dyadic junction (Louch et al., 2010). Labeling techniques capable of resolving the localized positions of LCC clusters would permit a more detailed analysis of LCC and NCX contributions to Ca^2+^ trigger flux (Jayasinghe et al., 2009).

### Allosteric catalysis of Na^+^-Ca^2+^ exchanger and Ca^2+^ trigger fluxes

A unique feature of the presented model was its ability to directly examine the role of catalytic Ca^2+^-binding sites on NCX in regulating local and global Ca^2+^ trigger fluxes. Here our studies indicate that upon depolarization, the non-allosteric NCX rapidly entered reverse mode and contributed a substantial constant inward flux during the entire simulation with 10 mM [Na^2+^]*_i_* [*I*_NCXglobal(0mV)_ ∼ 3 μM/s, *I*_NCXglobal(+50mV)_ ∼ 8 μM/s, *I*_NCXsarc(0mV)_ ∼ 9 μM/s, *I*_NCXsarc(+50mV)_ ∼ 22 μM/s]. At 0 mV, when the LCC flux was dominant, a modest decrease in *d*[Ca^2+^]_mouth_/*dt*_max_ (2.5 vs. 3 μM/s) was predicted. At +50 mV, when NCX contributes a significantly larger fraction of the total Ca^2+^ transient, the effects of NCX allostery were more evident. Assumption of a non-allosteric NCX model resulted in a approximately twofold drop in *d*[Ca^2+^]_mouth_/*dt*_max_ and lesser inwardly propagating Ca^2+^ gradient at +50 mV (Movie S3 in Supplementary Material *lower left panel* vs. Movie S1 in Supplementary Material *lower left panel*). These data reveal that the allosteric catalysis of NCX can augment the local trigger fluxes as well (Litwin et al., 1996; Ramirez et al., 2011). No visible changes, however, were detected in *d*[Ca^2+^]_end_/*dt*_max_ and *d*[Ca^2+^]_global_/*dt*_max_ at both applied voltages (*data not shown*). A possible reason for the negligible predicted effects of NCX allostery on *d*[Ca^2+^]_end_/*dt*_max_ and *d*[Ca^2+^]_global_/*dt*_max_, which is at odds with experimental estimates of the global trigger fluxes (Figure 2F), might be the assumed continuous NCX distribution along the cell membrane. We speculate that the NCX allosteric effect would be much more pronounced, if reverse-mode Ca^2+^ entry were localized to a smaller region typical of a cluster, which could then activate Ca^2+^ binding to NCX to a greater degree (Jayasinghe et al., 2009). Moreover, the relative spacing between an NCX cluster and LCC and alterations in normal NCX maximum rate and allosteric constant (Movie S5 in Supplementary Material) could modulate the amplitude of the NCX reverse mode.

### Limitations of the model

Although we demonstrate good correlation with whole-cell experimental data using a relatively small sub-cellular geometric domain, this assumption implies that all domains are identical in shape and flux distribution. T-tubule data in rabbits from Savio-Galimberti et al. (2008), however, suggest considerable variety both in terms of the diameter, shape, and arrangement of tubules, as well as the shape of the cell exterior. Including such details could permit investigation into coupling between adjacent tubules in rabbits.

Furthermore, we restrict our model to Ca^2+^ and Ca^2+^-buffer dynamics, whereas additional ions, namely Na^+^ and K^+^, play a significant role in modulating ECC coupling (Despa et al., 2003; Bers and Despa, 2009; Torres et al., 2010). In particular, our results suggest that changes in [Na^+^]*_i_* modulate NCX activity independent from any Ca^2+^-dependent allosteric effects (Movie S3 in Supplementary Material). By explicitly including the primary fluxes for Na^+^ and K^+^ (*I*_Na_, *I*_NaK_), intracellular Na^+^ diffusion and buffering, and distributions of Na^+^ channels and Na^+^/K^+^ pumps along the sarcolemma we may further examine the role of NCX allostery due to LCC opening, especially early in the AP. We further anticipate that the allosteric interaction between NCX and LCCs will be more nuanced during an AP, given that the fast Na^+^ promotes activation of the exchanger before many LCCs have opened (Torres et al., 2010). In this study also, the mitochondrial Ca^2+^ fluxes were omitted (Dash et al., 2009; Pradhan et al., 2010). These are likely to have important impact on predicted Ca^2+^ profiles as well (Nguyen et al., 2007; Cortassa and Aon, 2012; Dedkova and Blatter, 2012).

## Conclusion

We developed and validated a 3D reaction-diffusion model of Ca^2+^ signaling in rabbit ventricular myocytes. The model incorporates realistic t-tubule and cell surface topologies, as well as clusters of LCCs along the t-tubule membrane. The key findings are: (1) the linear t-tubule topology and the punctuate spatial distribution of LCC along the sarcolemma function to inhibit inwardly propagating Ca^2+^ gradients; (2) local trigger fluxes of Ca^2+^ are at least threefold to eightfold higher than whole-cell Ca^2+^ trigger flux; and (3) the activation of allosteric Ca^2+^-binding sites on Na^+^/Ca^2+^ exchanger could provide a mechanism that regulates local and global Ca^2+^ trigger fluxes *in vivo*. We concluded that improved models representing the localized positions and the functional diversity of LCC clusters could help to improve our understanding of *I*_LCC_ and *I*_NCX_ contributions to global and local Ca^2+^ trigger fluxes.

## Conflict of Interest Statement

The authors declare that the research was conducted in the absence of any commercial or financial relationships that could be construed as a potential conflict of interest.

## Supplementary Material

The Supplementary Material for this article can be found online at http://www.frontiersin.org/Computational_Physiology_and_Medicine/10.3389/fphys.2012.00351/abstract

Supplementary Movie S1 **Effects of voltage and intracellular Na^+^ on spatial Ca^2+^ dynamics in the presence of allosteric catalysis and 50 μM Fluo-3**.

Supplementary Movie S2 **Effects of continuous LCC current distribution along the t-tubule on spatial Ca^2+^ dynamics in the presence of allosteric catalysis, 50 μM Fluo-3 and 10 mM [Na^+^]*_i_***.

Supplementary Movie S3 **Effects of voltage and intracellular Na^+^ on spatial Ca^2+^ dynamics in the absence of allosteric catalysis and presence of 50 μM Fluo-3**.

Supplementary Movie S4 **Effects of LCC cluster placement along t-tubule on spatial Ca^2+^ dynamics at 0 mV in the presence of allosteric catalysis, 50 μM Fluo-3 and 10 mM [Na^+^]*_i_***.

Supplementary Movie S5 **Effects of changes in NCX allosteric constant and maximum NCX rate on spatial Ca^2+^ dynamics at +50 mV in the presence of allosteric catalysis, 50 μM Fluo-3 and 10 mM [Na^+^]*_i_***.

## Appendix

### Experimental and Simulation Protocols

#### Confocal imaging, post-processing, and geometric modeling

Myocytes were superfused with membrane-impermeant dextran conjugated to fluorescein and imaged with a BioRad MRC-1024 laser-scanning confocal microscope (Savio-Galimberti et al., 2008). Image data were devonvolved, corrected for background signals, and depth dependent attenuation. Single or groups of t-tubules were segmented using region-growing and stored together with an associated segment of outer sarcolemma. A modified Marching cube algorithm was used to create an initial triangulation from the segmented data (Heiden et al., 1993). A GAMer plugin to Blender was used to assign markers for the cell surface, t-tubule, and interior boundaries (Yu et al., 2008a). TetGen was then used to convert the surface mesh into a tetra-hedralized mesh suitable for finite-element solution (http://wias-berlin.de/software/tetgen/). The created geometric model contained realistic surface sarcolemma, realistic t-tubule, and its surrounding half-sarcomeres.

#### Currents and calcium concentration simulation protocols

The voltage-clamp protocols (holding potential −50 mV, voltage step to 0 or +50 mV for 200 ms) and whole-cell LCC currents were derived from data in rabbit ventricular myocytes with RyR Ca^2+^ release and SERCA Ca^2+^ uptake disabled pharmacologically (Sobie et al., 2008).

Model simulation protocols for [Ca^2+^]*_i_* resemble the protocols applied previously in experimental studies where the cells, incubated with ryanodine and thapsigargin, were loaded with 50 μM Fluo-3, and confocal line-scan recordings of fluorescence ratio (*F*/*F*_o_) reflecting the changes of Ca^2+^ concentration in time were made concurrently (Sobie et al., 2008). This allowed simultaneous measurements of whole-cell LCC current and the total transmembrane flux of Ca^2+^ under conditions promoting Ca^2+^ entry via NCX; positive potentials and [Na^+^]*_i_* of 10 mM Sobie’s et al. data indicate: (1) that at *V*_m_ ≥ 20 mV, *dF*/*dt*_max_ was larger with 10 mM [Na^+^]*_i_* than that measured with 0 mM [Na^+^]*_i_* (zero Na^+^ prohibits Ca^2+^ entry through NCX), suggesting that reverse-mode NCX augments Ca^2+^ trigger flux at positive voltages; and (2) that under control conditions (10 mM [Na^+^]*_i_* and *V*_m_ ≥ +30 mV) *dF*/*dt*_max_ is much greater than a simple sum of fluxes due to LCC current and reverse-mode NCX, suggesting possible activation of an allosteric NCX Ca^2+^-binding site.

### Model Parameters

**Table A1:** **Physical constants and cell geometry parameters**.

Symbol	Definition	Value	Reference
**PHYSICAL CONSTANTS**			
*F*	Faraday constant	96.5°C mmol^−1^	Physical constant
*T*	Temperature	295 K	Physical constant
*R*	Universal gas constant	8.314 J mol^−1^ K^−1^	Physical constant
**WHOLE-CELL GEOMETRY**			
*V*_cell_	Cell volume	30.4 pL	Satoh et al. (1996)
*C*_m_	Cell capacitance	138 pF	Satoh et al. (1996)
**COMPARTMENT AND t-TUBULE GEOMETRIES**			
*V*_ms_	Compartment volume	2.82e−2 pL	Estimated
*S*_mc_	Compartment surface	15.9 μm^2^	Estimated
*S*_mc-tubule_	T-tubule surface area	7.8 μm^2^	Estimated
*d_lng_*	Longitudinal cell direction	2.58 μm	Estimated
*d*_ax_	Axial cell direction	2.34 μm	Estimated
*d*_tr_	Transverse cell direction	5.76 μm	Estimated
*r*_tubule_	T-tubule radius	0.2–0.31 μm	Savio-Galimberti et al. (2008)
*l*_tubule_	T-tubule length	4.6 μm	Savio-Galimberti et al. (2008)

**Table A2:** **Calcium and buffer reaction-diffusion parameters**.

Symbol	Definition	Value	Reference
**Ca^2+^, Na^+^, AND BUFFER CONCENTRATIONS**			
[Ca^2+^]*_o_*	Extracellular Ca^2+^ concentration	2000 μM	Sobie et al. (2008)
[Ca^2+^]*_i0_*	Resting Ca^2+^ concentration	0.1 μM	Sobie et al. (2008)
[Na^+^]*_o_*	Extracellular Na^+^ concentration	140 mM	Sobie et al. (2008)
[Na^+^]*_i0_*	Resting Na^+^ concentration	10 mM	Sobie et al. (2008)
[TN]	Total troponin concentration	70 μM	Shannon et al. (2004)
[ATP]	Total free ATP concentration	260 μM	Cheng et al. (2010)
[CaM]	Total calmodulin concentration	24 μM	Shannon et al. (2004)
[Fluo]	Total Fluo-3 concentration	50 μM	Sobie et al. (2008)
**DIFFUSION CONSTANTS (AT 22°C)**			
*D*_ca_	Diffusion coefficient for Ca^2+^	0.39 μm^2^ ms^−1^	Cheng et al. (2010)
*D*_CaFluo_	Diffusion coefficient for CaFluo	0.1 μm^2^ ms^−1^	Cheng et al. (2010)
*D*_CaATP_	Diffusion coefficient for CaATP	0.168 μm^2^ ms^−1^	Cheng et al. (2010)
*D*_CaCam_	Diffusion coefficient for CaCaM	0.025 μm^2^ ms^−1^	Cheng et al. (2010)
**RATE COEFFICIENTS AND DISSOCIATION CONSTANTS (AT 22°C)**			
$k_{\text{ + }}^{\text{CaTN}}$	Ca^2+^ on-rate constant for TN	0.04 μM^−1^ ms^−1^	Shannon et al. (2004)
$k_{\text{ + }}^{\text{CaTN}}$	Ca^2+^ off-rate constant for TN	0.04 ms^−1^	Shannon et al. (2004)
$k_{\text{D}}^{\text{TN}}$	Ca^2+^ dissociation constant for TN	1 μM	Shannon et al. (2004)
$k_{\text{ + }}^{\text{CaATP}}$	Ca^2+^ on-rate constant for CaATP	0.225 μM^−1^ ms^−1^	Cheng et al. (2010)
$k_{-}^{\text{CaATP}}$	Ca^2+^ off-rate constant for CaATP	45 ms^−1^	Cheng et al. (2010)
$k_{D}^{\text{ATP}}$	Ca^2+^ dissociation constant for ATP	200 μM	Cheng et al. (2010)
$k_{\text{ + }}^{\text{CaCaM}}$	Ca^2+^ on-rate constant for Cal	0.125 μM^−1^ ms^−1^	Shannon et al. (2004)
$k_{-}^{\text{CaCaM}}$	Ca^2+^ off-rate constant for Cal	0.2975 ms^−1^	Shannon et al. (2004)
$k_{\text{D}}^{\text{CaM}}$	Ca^2+^ dissociation constant for Cal	2.38 μM	Shannon et al. (2004)
$k_{\text{ + }}^{\text{CaFluo}}$	Ca^2+^ on-rate constant for CaFluo	0.23 μM^−1^ ms^−1^	Cheng et al. (2010)
$k_{-}^{\text{CaFluo}}$	Ca^2+^ off-rate constant for CaFluo	0.17 ms^−1^	Cheng et al. (2010)
$k_{\text{D}}^{\text{Fluo}}$	Ca^2+^ dissociation constant for Fluo	0.739 μM	Cheng et al. (2010)

**Table A3:** **Membrane calcium fluxes parameters**.

Symbol	Definition	Value	Reference
**L-TYPE Ca^2+^ CURRENT**			
*a*_(0mV)_	Constant	3.28e−08	Estimated
*b*_(0mV)_	Constant	−1.388e−05	Estimated
*c*_(0mV)_	Constant	0.002	Estimated
*d*_(0mV)_	Constant	−0.136	Estimated
*e*_(0mV)_	Constant	3.711	Estimated
*a*_(+50mV)_	Constant	3.366e−09	Estimated
*b*_(+50mV)_	Constant	−1.426e−06	Estimated
*c*_(+50mV)_	Constant	0.000	Estimated
*d*_(+50mV)_	Constant	−0.019	Estimated
*e*_(+50mV)_	Constant	0.981	Estimated
β_Ca_	Scaling constant	28.5	Estimated
**Na^+^/Ca^2+^ EXCHANGE CURRENT**			
*V*_max_NCX_	Maximum NCX rate	0.207 μM ms^−1^	Shannon et al. (2004)
*K*_mCaAct_	Allosteric constant	0.29 μM	Estimated
$K_{\text{mC}{\text{a}}_{o}}$	Extracellular Ca2^+^ dissociation constant	1.3e−3 μM	Shannon et al. (2004)
$K_{\text{mC}{\text{a}}_{i}}$	Intracellular Ca2^+^ dissociation constant	3.59 μM	Shannon et al. (2004)
$K_{\text{mN}{\text{a}}_{o}}$	Extracellular Na^+^ dissociation constant	87.5e−3 μM	Shannon et al. (2004)
$K_{{\text{mNa}}_{i}}$	Intracellular Ca2^+^ dissociation constant	12.29e−3 μM	Shannon et al. (2004)
η	Voltage-dependent factor	0.35	Shannon et al. (2004)
*k*_sat_	Low potential saturation factor	0.27	Shannon et al. (2004)
β_NCX_	Scaling constant	28.5	Estimated
**SARCOLEMMAL Ca^2+^ ATPase PUMP**			
*V*_max_Pump_	Maximum pump rate	2.2e−3 μM ms^−1^	Shannon et al. (2004)
*K*_mPump_	Half-saturation constant	0.5 μM	Shannon et al. (2004)
*n*_Hill_	Hill coefficient	1.6	Shannon et al. (2004)
β_Pump_	Scaling constant	28.5	Estimated
**SARCOLEMMAL Ca^2+^ LEAK**			
$V_{\max\_\text{Leak}}^{10\text{mM}{\left[{\text{Na}}^{+}\right]}_{i}}$	Conductance	0.001984 μM ms^−1^	Estimated
$V_{\max\_\text{Leak}}^{0\text{mM}{\left[{\text{Na}}^{+}\right]}_{i}}$	Conductance	0.004382 μM ms^−1^	Estimated
β_Leak_	Scaling constant	28.5	Estimated

#### Model equations

##### Reaction-diffusion equations

$$\begin{array}{llll} & \frac{\partial {\left[\text{C}{\text{a}}^{2+}\right]}_{i}}{\partial t}=D_{\text{Ca}}\nabla^{\text{2}}{\left[\text{C}{\text{a}}^{2+}\right]}_{i}-\overset{3}{\underset{m=1}{\sum }}R_{B_{\text{m}}}-R_{B_{\text{s}}}+J_{\text{C}{\text{a}}_{\text{flux}}} & & \text{(A1)}\\ & \frac{\partial \left[\text{Ca}B_{\text{m}}\right]}{\partial t}=D_{\text{Ca}B_{\text{m}}}\nabla^{\text{2}}\left[\text{Ca}B_{\text{m}}\right]+R_{B_{\text{m}}} & & \text{(A2)}\\ & \frac{\partial \left[\text{Ca}B_{\text{s}}\right]}{\partial t}=R_{B_{\text{s}}} & & \text{(A3)}\\ & R_{B_{\text{m}}}=k_{+}^{m}\left(\left[B_{\text{m}}\right]-\left[\text{Ca}B_{\text{m}}\right]\right){\left[\text{C}{\text{a}}^{2+}\right]}_{i}-k_{-}^{m}\left[\text{Ca}B_{\text{m}}\right] & & \text{(A4)}\\ & R_{B_{\text{s}}}=k_{+}^{s}\left(\left[B_{\text{s}}\right]-\left[\text{Ca}B_{\text{s}}\right]\right){\left[\text{C}{\text{a}}^{2+}\right]}_{i}-k_{-}^{s}\left[\text{Ca}B_{\text{s}}\right] & & \text{(A5)} \end{array}$$

where [*B*_m_] represents the concentration of mobile buffer Fluo-3, ATP, or calmodulin; [*B*_s_] is the concentration stationary buffer troponin C.

In the model we assume: (1) isotropic diffusion for Ca^2+^ and all mobile buffers (Soeller et al., 2009); (2) Ca^2+^ binds to Fluo-3, calmodulin, ATP, and TN without cooperativity; (3) the initial total concentrations of the mobile buffers are spatially uniform; (4) the diffusion coefficients of Fluo-3, ATP, or calmodulin with bound Ca^2+^ are equal to the diffusion coefficients of free Fluo-3, ATP, or calmodulin.

#### Model currents

The total Ca^2+^ flux across the t-tubule and external membrane is described as:

(A6) $$J_{\text{C}{\text{a}}_{\text{flux}}}=J_{\text{LCC}}+J_{\text{NCX}}+J_{\text{Pump}}+J_{\text{Leak}}$$

Each current density was converted to Ca^2+^ flux by using the experimentally suggested surface to volume ratio (*C*_m_/*V*_cell_∼4.54 pF/pL) in adult rabbit ventricular myocytes (Satoh et al., 1996; Bers, 2001):

(A7) $$J_{i}=\beta_{i}\left(\frac{1}{2F}\frac{C_{\text{m}}}{V_{\text{cell}}}\frac{V_{\text{ms}}}{S_{\text{ms}}}\right)I_{i}$$

where *V*_ms_ and *S*_ms_ are total surface and volume of the real or ideal model compartment and β*_i_* scaling constant.

##### L-type Ca^2+^ current (*I*_LCC_)

L-type Ca^2+^ current density was parameterized to fit the current profiles recoded at 0 mV and +50 mV (Sobie et al., 2008):

$$\begin{array}{rcll} & I_{\text{LCC(0}\;\text{mV)}}\left(t\right)=a_{\left(0\;\text{mV}\right)}t^{4}+b_{\left(0\;\text{mV}\right)}t^{3}+c_{\left(0\;\text{mV}\right)}t^{2} & & \\ & +\;d_{\left(0\;\text{mV}\right)}t+e_{\left(0\;\text{mV}\right)} & & \text{(A8)}\\ & I_{\text{LCC( + 50}\;\text{mV)}}\left(t\right)=a_{\left(+50\;\text{mV}\right)}t^{4}+b_{\left(+50\;\text{mV}\right)}t^{3}+c_{\left(+50\;\text{mV}\right)}t^{2} & & \\ & +\;d_{\left(+50\;\text{mV}\right)}t+e_{\left(+50\;\text{mV}\right)} & & \text{(A9)} \end{array}$$

##### Na^+^/Ca^2+^ exchanger current (I_NCX_)

The steady-state relation between [Ca^2+^]*_i_* and *I*_NCX_ is described as in Weber et al. (2001):

(A10) $$I_{\text{NCX}}=\text{Allo}\times \text{Bind}\times \text{Elect}$$

$$\begin{array}{llll} & \text{Allo}=\frac{V_{\max\text{\_}\text{NCX}}}{1+{\left(\frac{K_{\text{mCaAct}}}{{\left[\text{C}{\text{a}}^{\text{2 + }}\right]}_{i}}\right)}^{3}} & & \text{(A11)} \end{array}$$

(A12) $$\text{Bind}=\frac{1}{\left\{\begin{array}{c} K_{{\text{mCa}}_{o}}{\left[{\text{Na}}^{+}\right]}_{i}^{3}+K_{{\text{mNa}}_{o}}{\;}^{3}{\left[{\text{Ca}}^{2+}\right]}_{i}+K_{{\text{mNa}}_{i}}{\;}^{3}{\left[{\text{Ca}}^{2+}\right]}_{o}\\ \left(1+\frac{{\left[{\text{Ca}}^{2+}\right]}_{i}}{K_{{\text{mCa}}_{\text{i}}}}\right)+K_{{\text{mCa}}_{i}}{\left[{\text{Na}}^{+}\right]}_{o}^{3}\left(1+\frac{{\left[{\text{Na}}^{+}\right]}_{i}^{3}}{K_{{\text{mNa}}_{\text{i}}}3}\right)+\\ {\left[{\text{Na}}^{+}\right]}_{i}^{3}{\left[{\text{Ca}}^{2+}\right]}_{o}+{\left[{\text{Na}}^{+}\right]}_{o}^{3}{\left[{\text{Ca}}^{2+}\right]}_{i} \end{array}\right\}}$$

(A13) $$\text{Elect}=\frac{{\left[{\text{Na}}^{+}\right]}_{i}^{3}{\left[{\text{Ca}}^{2+}\right]}_{o}{\text{e}}^{\eta VF/RT}-{\left[{\text{Na}}^{+}\right]}_{o}^{3}{\left[{\text{Ca}}^{2+}\right]}_{i}{\text{e}}^{\left(\eta -1\right)VF/RT}}{1+k_{\text{sat}}{\text{e}}^{\left(\eta -1\right)VF/RT}}$$

The three factors are: Allo-allosteric regulation by [Ca^2+^]*_i_*, which is not transported; Bind-competitive binding of Na^+^ and Ca^2+^ as substrates; Elect-effects of membrane potential. The latter two combined give an electrochemical term, i.e., Δ*E* = Bind × Elect.

##### Sarcolemmal Ca^2+^ ATPase pump (*I*_Pump_)

The equation and describing *I*_Pump_ dependence on [Ca^2+^]*_i_* is from Shannon et al. (2004):

(A14) $$I_{\text{Pump}}=\frac{V_{\max}-\text{Pump}}{1}+{\left(\frac{K_{\text{m}\;\text{Pump}}}{{\left[\text{C}{\text{a}}^{2+}\right]}_{i}}\right)}^{n_{\text{Hill}}}$$

##### Sarcolemmal Ca^2+^ leak (*I*_Leak_)

The equation for *I*_Leak_ dependence on [Ca^2+^]*_i_* is as in Shannon et al. (2004):

(A15) $$I_{\text{Leak}}=V_{\max-\text{Leak}}\left[V-\frac{RT}{\text{2}F}\ln\left(\frac{{\left[\text{C}{\text{a}}^{2+}\right]}_{o}}{{\left[\text{C}{\text{a}}^{2+}\right]}_{i}}\right)\right]$$

At rest the Ca^2+^ influx via background Ca^2+^ leak is adjusted to match the Ca^2+^ efflux via NCX and Ca^2+^ ATPase so that no net movement across the cell membrane to occurred.

### Numerical Algorithms and Software

In finite-element methods, a complex domain needs to be discretized into a number of small elements (such as triangles or tetrahedra). This process is usually referred to as mesh generation (Yu et al., 2008a,b). Although different types of meshes may be generated depending on the numerical solvers to be employed, we restrict ourselves to triangular (surface) and tetrahedral (volumetric) mesh generation as commonly used in biomedical simulation. In the present simulation, the number of finite-element nodes and tetrahedral elements are 4274 and 13,673, respectively.

The non-linear reaction-diffusion system was solved by a finite difference method in time and finite-element method in space using our SMOL software tool (Smoluchowski Solver, http://mccammon.ucsd.edu/smol/) with the time step of 0.5 ms. It takes around 60 min to run 200 ms snapshots with a single Intel Xeon X5355 processor. The SMOL program utilizes libraries from the finite-element tool kit (FETK), which previously has been used in several molecular level studies (Cheng et al., 2007a,b, 2008). One bottleneck for dynamic 3D simulation of non-linear reaction-diffusion system is the computing complexity involved in solving the problem. Here we successfully extended SMOL to solve multiple coupled partial differential equations with non-linear ordinary equations. Multiple tests demonstrate that our SMOL program is quite robust and flexible for various boundary and initial conditions. The simulation results were visualized using GMV mesh viewers (Ortega, 1996). Post-processing and data analyses were implemented by customized Python, MATLAB 2008b (The MathWorks, Natick, MA, USA) scripts and Xmgrace software (Vaught, 1996). A version control system, subversion, was used to monitor the development of software (Collins-Sussman et al., 2002).
